# Multi-Layer Filters: Adsorption and Filtration Mechanisms for Improved Separation

**DOI:** 10.3389/fchem.2018.00417

**Published:** 2018-09-12

**Authors:** Aysu Onur, Aaron Ng, Warren Batchelor, Gil Garnier

**Affiliations:** ^1^Chemical Engineering Department, Bioresource Processing Research Institute of Australia, Monash University, Clayton, VIC, Australia; ^2^3M Australia, Sydney, NSW, Australia

**Keywords:** composite, multi-layer, adsorption, filtration, cellulose fiber, channeling

## Abstract

Filters made of cellulose fiber and perlite particles were prepared using a wet laying papermaking technique. Polyamide-amine-epichlorohydrin (PAE) was added to provide wet strength. Filters were prepared at two different total basis weights of 200 and 400 grams per square meter (gsm). Single and multi-layered filters were structured for each total basis weights. The effect of total basis weights and multi-layered structure on methylene blue adsorption and silicon dioxide (SiO_2_) particle filtration was investigated. Methylene blue adsorption was performed in two modes: constant pressure and constant flow rate. In both operation modes, the adsorption capacity of multi-layered filters was significantly higher (16–100%) than for single-layer filters at the same overall weight. The effect of layer separation was also characterized using polypropylene separators and tested under constant flow rate operation. Separators provided more effective methylene blue adsorption by generating a well-distributed flow. Filtration performance was quantified with 0.5 μm silicon dioxide particles under constant pressure conditions; this is to mimic bacteria rejection. Filtration capability of SiO_2_ particles was reduced slightly (12%) with decreasing individual filter layer thickness regardless of the multi-layered structure. Filtering polyethylene glycol (PEG) molecules with two different molecular weights was performed; however, no rejection was recorded. The filter internal pore structure was visualized by 3D-X ray computed tomography and the void fraction was quantified. 400 gsm single layer presented areas of low fiber density forming pores, while the pore volume decreased for thinner filter layers.

## Introduction

Depth filters (Sutherland, 2011) are porous filtering mediums composed of cellulose fibers and inorganic absorbents. Unlike surface filtration, they can retain contaminants through the thickness as well as on the surface during liquid filtration. These composite structures combine two different separation principles and technologies in a single medium. Filtration by particle rejection is provided by forming an intricate mesh, where selective adsorption is achieved by a functional inorganic particle. Separation can further be improved by combining other additives, such as charged polyelectrolytes in the network (Dizge et al., 2011). The medium can be modified with a cationic polymer adsorbing the common negatively charged dissolved contaminants significantly smaller than the average pore size. However, depth filtration analysis reported in literature has mostly focused on the modeling of membrane separations (Polyakov, 2008, 2009; Sutherland, 2011; Kuhn and Briesen, 2016; Bedrikovetsky et al., 2017; Goldrick et al., 2017); there is a lack of experimental studies optimizing depth type filter operation especially in terms of membrane structure.

Filtration performance can be tailored in many ways by controlling filter structure/composition and operation mode. Two efficient modes of operation are dead-end filtration and cross-flow filtration, where the flow is passed directly through the filter in dead-end and tangential to the filter in cross-flow filtration (Liderfelt and Royce, 2018), respectively. Furthermore, these processes can be controlled under modes of constant pressure or constant flow rate. In either case, the non-constant parameter is being monitored, and the extent of filter fouling or clogging is assessed by the recorded parameters (Iritani et al., 2015; Goldrick et al., 2017). Filtration performance can also be altered by modifying the structure and configuration of filters. Multi-layer structured filters with varying pore sizes stacked on top of another offer a simple way of sequentially separating cells or particles (Saefkow, 1995; Rijn, 1998). In a recent study by Griffiths et al. (Griffiths et al., 2016), filtration was modeled on a multi-layered membrane structure with each layer having varying pore sizes stacked on one another. The efficiency of multi-layered filter structure was analyzed by developing a model simulating the transport and filtration of particles through a multi-layer structure. This model characterizes the filter and offers optimal design requirements in terms of number of filter layers, pore size in each layer and pore interconnectivity between layers.

Filtering bacteria with multi-layered filters to provide a greater capture of bacteria was also reported (Koch, 1984). The study aimed at achieving higher bacteria rejection by mechanically attaching a layer to another, which contains a bacteria-destroying material. In another study (Wertz and Guimond, 2014), a nano fiber layer was attached to the initial layer of a fibrous filter. Filter media having a first layer and a nanofiber layer adhered onto exhibited advantageous properties including increased dust holding capacity.

Although multi-layered filters are well-known in industry, very few studies have systematically quantified their adsorption and filtration mechanisms, and even less have characterized the effect of multi-layered structure on depth filter performance. In this study, we developed multi-layered and single-layered filter structures made from the same amount of filter media. Depth filter layer processing was inspired from papermaking technique and characterized for adsorption and particle rejection capability. The adsorption and filtration mechanisms behind the performance of single and multi-layered filters were analyzed in terms of chemical engineering and internal filter structure using an advanced image technique and colloids and surface concepts.

## Materials and methods

### Materials

Cellulose fibers used for the composites are unrefined northern softwood NIST RM 8495 bleached Kraft pulp and bleached radiata pine softwood Kraft pulp refined to 400 Canadian standard freeness (CSF) in a disk refiner. Expanded perlite was provided by Dicalite Minerals Corp. Commercial PAE was provided from Nopcobond Paper Technology Pty Ltd.

Methylene blue (MB) in powder form and 0.5 μm monodispersed silicon dioxide (SiO_2_) particles of density 1.8 g/cm^3^ were purchased from Sigma Aldrich. Polyethylene glycol (PEG) of two molecular weights (600 and 5,000 kDa) was provided by Dow Chemical. Polypropylene separators with 0.45 μm pore size and diameter of 47 mm was purchased from CUNO Inc. Meriden, USA.

### Methods

#### Fabrication of filters

**Figure 1:**
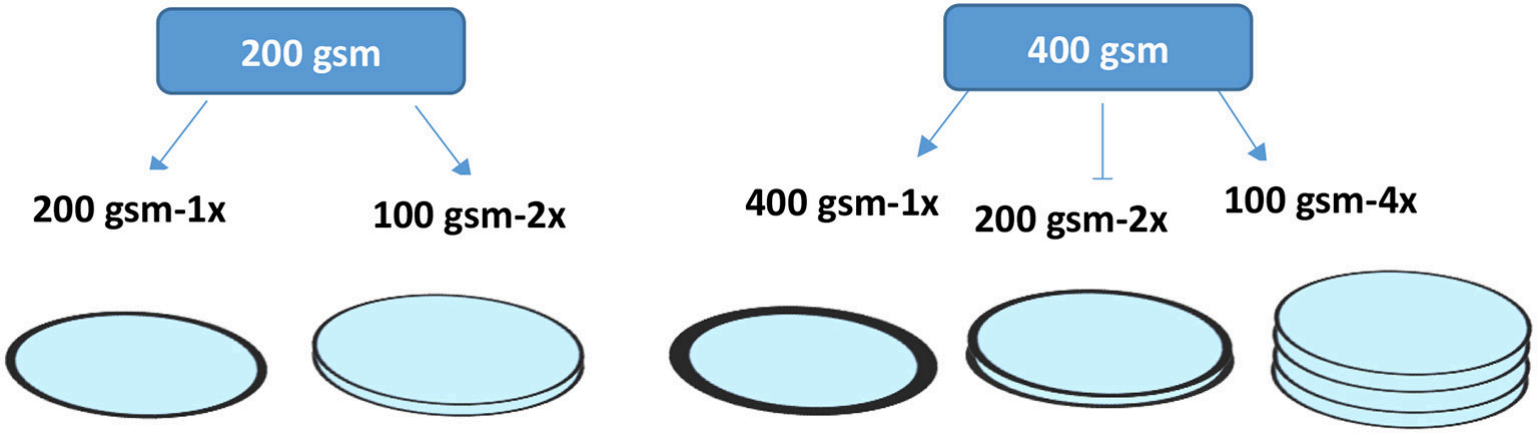
Schematic representation of the different filter configurations investigated at two different basis weights.

The dry northern softwood NIST pulp was wetted by soaking in deionized water overnight. The pulp was transferred to a disintegrator (Model MKIIIC, Messmer Instruments Ltd.) and disintegrated for 75,000 revolutions. Filters were prepared using a standard British hand sheet maker at three different total basis weights: 100, 200, and 400 gsm. The step by step preparation method was published in a previous study (Onur et al., 2018). The filter chemical composition is similar to that of standard industrial filters: it consists of 30% fiber (1/3 refined pulp + 2/3 unrefined pulp) and 70% perlite with PAE (0.22% w/v) added at a rate of 100 mg/g fiber on top of the suspension (0.26 wt.%) before papermaking. Composites were prepared either as one single layer or in the form of stacked equal multilayers that is equivalent to the total targeted basis weight. Five different filters prepared according to different layer configurations and different total basis weights are shown in Figure 1. Samples were coded with the corresponding labels. The criteria for material selection are based on industrial filters for food and beverage applications; food grade filters usually contain these materials.

PAE is used to provide wet strength to the filters. It crosslinks with the carboxyl group of cellulose to create an irreversible covalent bond network and provides some waterproof barrier around fibers (Obokata and Isogai, 2007). PAE also adsorbs onto the fibers and particles of the suspension which introduces positive charges to the anionic surfaces of cellulose fibers and perlite particles. Addition of PAE to the suspension results in the partial surface coverage of perlite and cellulose fibers by positively charged polymer. This induces electrostatic interactions between negatively charged contaminants and the filter medium. Using PAE is allowed by food regulations at low concentrations (up to 2.5 wt.% of the dry sheet); it is considered an indirect food additive and the concentration of PAE of this study is around this limit (CFR–Code of Federal Regulations Title^1^).

Perlite is a low cost adsorbent made from a glassy volcanic rock; it is known as an excellent filter aid (Dogan et al., 2000). Perlite is an anionic adsorbent of zeta potential ranging between –40 and –50 mV (Alkan et al., 2005). The particle size distribution of perlite and its effect on the structure and porosity of filters was reported in a previous study (Onur et al., 2018).

#### Adsorption and filtration with constant pressure

Adsorption and filtration experiments under constant pressure mode were performed with a dead-end stirred cell from Merck Millipore Australia. The closed setup has 400 mL of working volume and is pressurized by a compressed air gas cylinder. Samples were placed on the membrane holder at the bottom of the cell body with an O-ring. For multilayers, the O-ring pressed layers from the edges so the interlayer gap was negligible. The cell body was filled with the desired solution following sample placement. Filtrate that passes through filters was transferred to an ultraviolet-visible (UV–Vis) absorption spectrometer via a quartz micro flow cell with 10 mm optical path length. Instantaneous absorbance was measured here every 10 s at a specific wavelength. After measurement in UV–Vis, filtrate was transferred to a beaker on a balance. Before conducting any experiments, water was flushed through samples to ensure equilibration. After equilibrium has been reached, either 5 ppm MB solution or 0.05 wt.% SiO_2_ suspension were passed through the filters at 1.5 bar. Two replicates of filters were tested for both adsorption and filtration. Here, *adsorption* corresponds to the accumulation of model cationic or anionic molecules onto a surface by electrostatic attraction, while *filtration* is measured as the rejection of particles in suspension by size exclusion. MB preferentially adsorbs onto negatively charged surfaces; it can also diffuse through the pores of the negatively charged perlite particles. However, there is no electrostatic attraction between MB molecules and the positively charged filter medium after adsorption of the PAE cationic polymer. MB was therefore selected as standard dye to quantify improvement in breakthrough curves due to the multi-layered filter structure as unable to adsorb.

Mass flux for the effective membrane area (0.00418 m^2^) was calculated by measuring the volume of permeate per unit area and time; Liter per Square Meter per Hour (LMH). Mass flow rate of filtrate as a function of time was also continuously recorded by a computer. Total flux was calculated with Equation (1).

(1) $$\begin{array}{l} FLUX\;\left(LMH\right)=\frac{Volume\;offiltrate\;\left(L\right)}{area\left(m^{2}\right)\;\times \;time\;\left(h\right)} \end{array}$$

Adsorption experiments were run with 400 mL MB dye solution at a concentration of 5 ppm at pH 5.8 (This is the characteristic pH of MB). Breakthrough curves were plotted by normalized concentrations as a function of time. Additional solution was added for filters that did not reach saturation with 400 mL solution. Area over the curve was calculated for each filter to determine the adsorption capacity of filters. Capacity was calculated based on Equation (2) (Barros et al., 2013). The integral was calculated from the area over the curve using the trapezoidal method.

(2) $$\begin{array}{l} q=\frac{C_{F}\times \dot{Q}}{1,000\times m_{s}}\int_{0}^{t_{end}}\left(1-\frac{C_{out}}{C_{F}}\right)dt \end{array}$$

Where:

*q*: Capacity of the adsorbent (mg/g)
*C*_*out*_*:* Concentration of MB in the filtrate (mg/g)
*C*_*F*_*:* Concentration of MB in the feed (mg/g)
$\dot{Q}$*:* Volumetric flow rate (cm^3^/s)
*t*_*end*_*:* Time (s)
*m*_*s*_*:* Weight of adsorbent (g)

Rejection of 0.5 μm SiO_2_ particles was determined by filtering 0.05 wt.% SiO_2_ suspension at the same pH value as dye molecules (pH = 5.8) through the filters. pH of 5.8 is desirable as increasing pH would dissociate carboxylic acids leading to repulsive forces between charged groups. These repulsive forces also lead to swelling of the cellulose fiber with collapse of the filter pore structure. SiO_2_ concentration of suspension before and after filtration was estimated by UV–Vis absorption spectroscopy and the Equation (3) was used to calculate rejection capability of filters. UV–Vis spectroscopy calibration curves of MB dye molecules and SiO_2_ particles are shown in the Supplementary information (Figure S5).

(3) $$\begin{array}{l} Rejection\;\left(\%\right)=1-\left(\frac{\text{Filtrate concentration}}{\text{Initial feed concentration}}\right)\times 100 \end{array}$$

PEG molecules were also filtered through the composites to quantify their microfiltration capability. The MWCO range for microfiltration applications usually starts from 500 kDa up to big particle filtration such as yeast and bacteria; the filtration spectrum can be found in the study (Pearce, 2007). Total carbon analyser was used to measure the concentration of PEG molecules in the suspension before and after filtration.

#### Adsorption with constant flow rate

**Figure 2:**
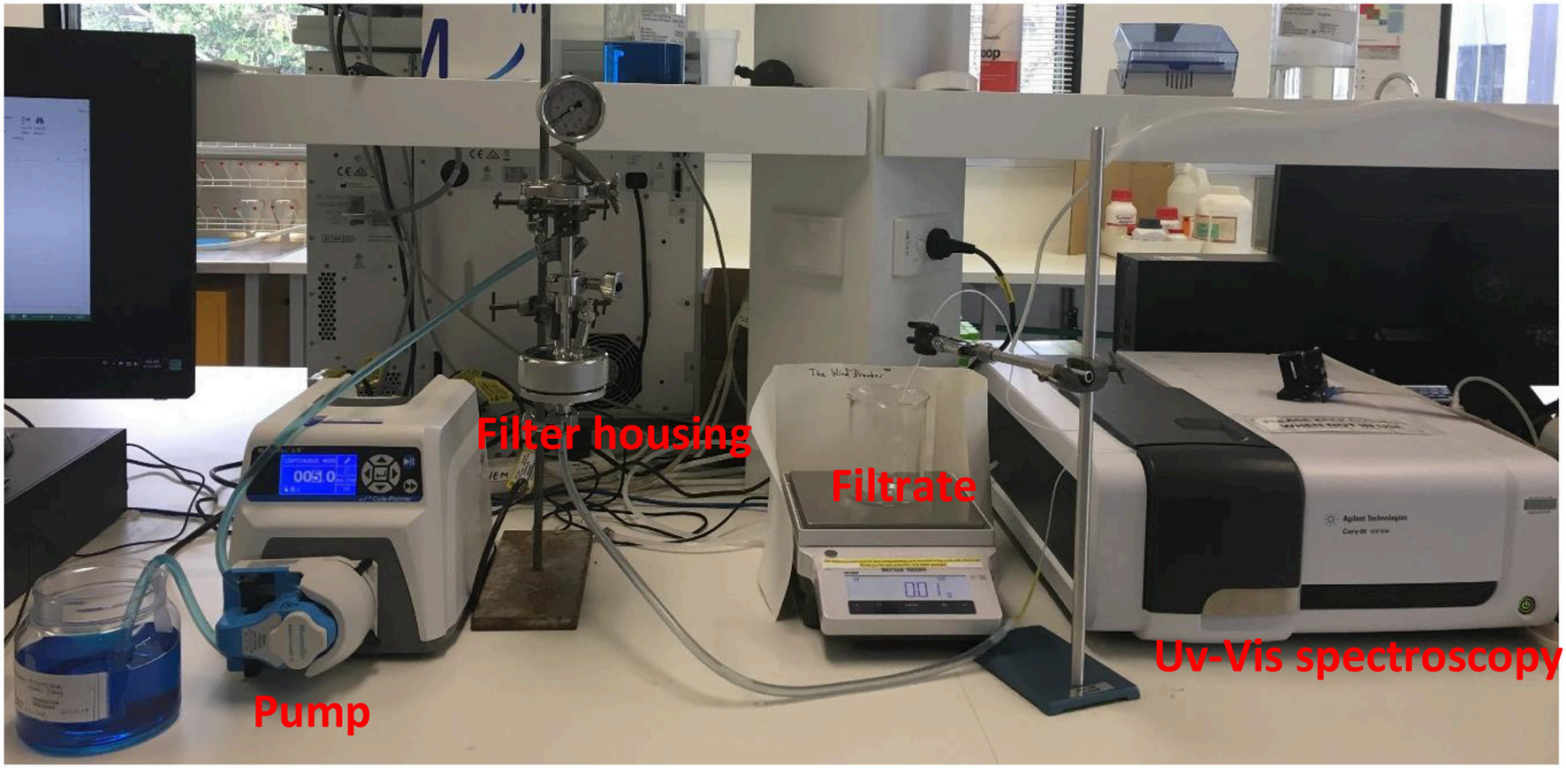
Experimental setup shown for 47 mm filter housings under constant flow rate mode.

Adsorption performance was additionally tested using 47 mm (corresponds to diameter of filter housings) stainless steel filter housings with 0.00132 m^2^ effective membrane area to verify the performance under constant flow rate operation mode. A peristaltic pump was used at a constant flow rate (12 mL/min) such that the same total flux as the constant pressure experiments for the same sample was obtained. Experiments were performed with and without presence of polypropylene separators. Separators are used to provide a well-distributed and uniform flow through a filter; they do not adsorb MB. Separators were placed in the housings as previously described (Adsorption and Filtration with Constant Pressure). The only gap created between multilayers is the thickness of the separator which is well below 1 mm. 47 mm separators were placed between the layers as well as on top of initial layers. Filtrate was transferred to UV-Vis spectroscopy via quartz micro flow cell to obtain the concentration at a regular time interval. Total mass flux and flow rate as a function of time were recorded by the same setup as constant pressure mode. Adsorption capacities were calculated form the experimental breakthrough curves as explained above. The experimental setup for constant flow rate operation is shown in Figure 2.

#### 3D X-ray tomography

X-ray computed 3D tomography was used to make a quantitative and qualitative analysis on the internal structure of filters about how the fiber network differently built up to form a sheet according to different basis weights. This technique provides a non-destructive 3D imaging. The basic principle is based on the set of images of beam transmitted through the sample while the sample is rotated to different positions for each image taken (Holmstad, 2004). Samples with 2 × 8 mm of size were visualized by Zeiss Xradia 520 Versa. The X-ray source was operated at 30 kV. Distance from source to sample and sample to detector was set to 15 mm. The number of images taken per scan was 1,601 and the image resolution was 2,022 × 2,022 pixels. Avizo and Image J software were used to reconstruct images and to quantify the total void fraction and void fraction distribution through the thickness (SI). Thresholding is done by comparing grayscale and thresholded image with a judgment. The threshold was meticulously selected with trial and error such that we do not lose any materials. Detailed information on scanning settings can be found in Supplementary materials (Figure S6). Void structure and how the void structure is changing through the thickness were analyzed qualitatively as well.

## Results

Cellulosic membranes of two different thicknesses (200 and 400 gsm) were tested under different filter configurations. The filters were examined under two operating conditions: at a constant flow rate (12 mL/min) or at a constant pressure (1.5 bar). The effect of polypropylene separators between the layers on adsorption performance was tested with 400 gsm single layer (400 gsm-1x) and four layers of 100 gsm filters (100 gsm-4x) under constant flow operation. Filtration of 0.1 wt.% PEG molecules of molecular weight of 600 and 5,000 kDa was performed under constant pressure (1.5 bar) mode for each filter configuration. Filters were also tested with suspensions of 0.5 μm SiO_2_ particles (0.05 wt.%) under constant pressure (1.5 bar). Finally, the internal structure of 400 gsm filters made of single and multiple layers was analyzed by 3D X-ray tomography combined with image analysis.

### Methylene blue adsorption under constant pressure

**Figure 3:**
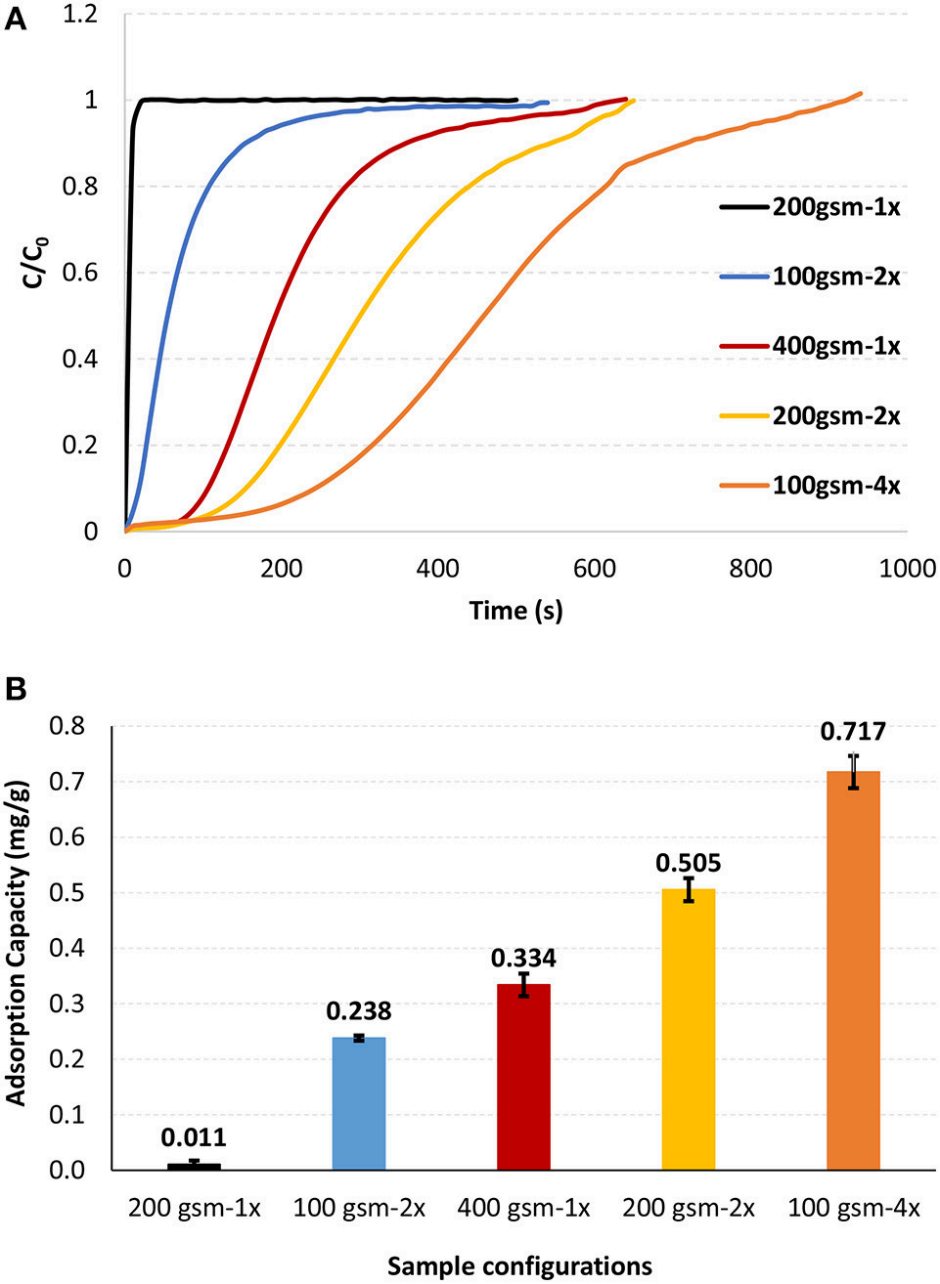
Effect of filter configuration and number of interfaces on the **(A)** methylene blue breakthrough curves and **(B)** methylene blue adsorption capacities. Tests were performed at 1.5 bar and 22°C.

Methylene blue was used as a model for the cationic organic dissolved contaminants. Constant pressure adsorption testing identifies filter compaction and saturation by measuring flowrate. Breakthrough curves were recorded under constant pressure (1.5 bar) for different filter configurations using two types of cellulose filters differing in thickness (gsm; Figure 3A). The adsorption capacity for each configuration was calculated using the breakthrough curves. The adsorption capacity of each filter is shown in Figure 3B.

Multilayer filters provided improved breakthrough curves and higher adsorption capacities than single layer filters as tested under constant pressure (Figure 3). Adsorption capacity of 400 gsm filter drastically increased by 54% and 118% by forming two layers (200 gsm-2x) and four layers (100 gsm-4x), respectively (Figure 3B), all at the same filter weight. Adsorption capacity of 200 gsm filter also increased significantly by forming two layers of 100 gsm (100 gsm-2x).

**Figure 4:**
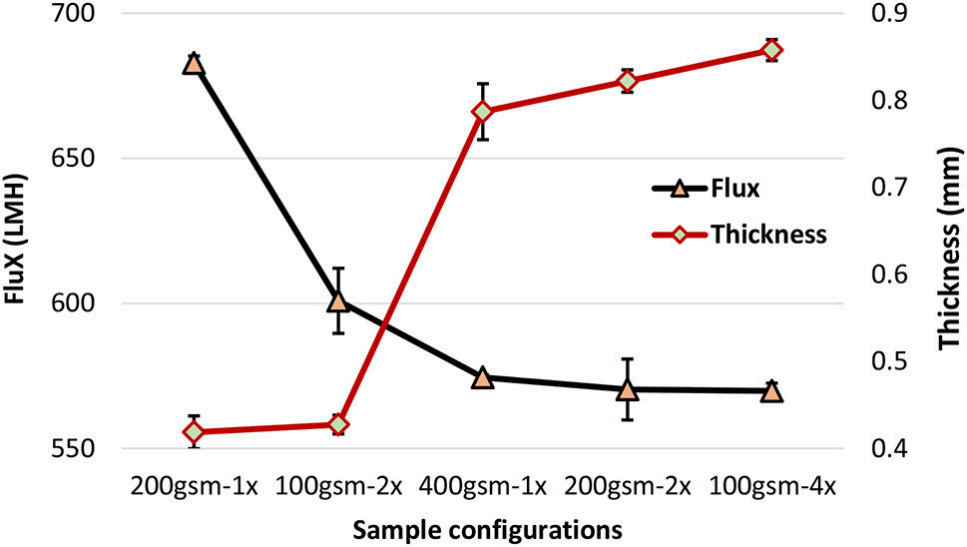
Effect of filter configuration and number of interfaces on the flux and thickness under 1.5 bar, 22°C.

The effect of filter configuration, including number and basis weight of layers, on filtration flux and filter thickness is shown in Figure 4 under constant pressure mode. At a given basis weight or amount of filter media, filter thickness increases slightly with multi-layered configuration. Flux decreased with increased gsm and the number of layers at a given gsm. Flux decreased from 682 to 600 LMH (Liters per Square Meter per Hour) for 200 gsm filters by introducing two layers of 100 gsm. However, flux slightly dropped for 400 gsm filters from 574 to 569 LMH for four layers of 100 gsm. For multi layered filters, an increased flux is expected according to Kozeny-Carman and Darcy (Siegfried Ripperger, 2000). These equations provide the mathematical relationships describing the flow of a fluid in a uniform porous media. Pressure loss between the layers might lead the flow traveling in the planar direction to find the path of least resistance through the next layer, resulting in an increased flux. However, this analysis does not fully apply to the space between layers. Once the stirred cell is pressurized by compressed air, the layers are being compressed and the flow is somewhat forced to flow through the next layer with little chance to flow in the planar direction. We believe that a slightly thicker layered structure would create a resistance and result in lower flux values.

### Methylene blue adsorption under constant flow rate

**Figure 5:**
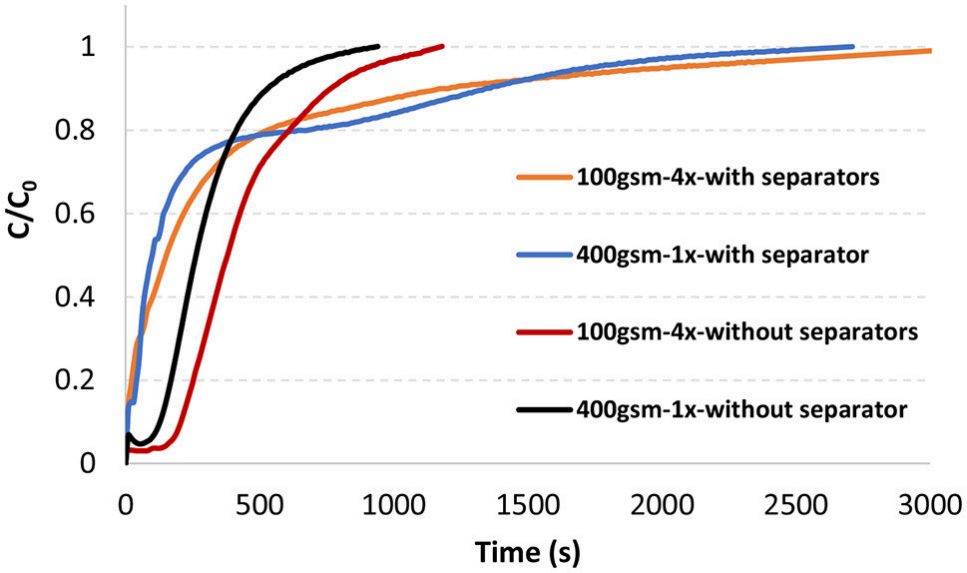
Methylene blue breakthrough curves of 47 mm 100 gsm-4x and 400 gsm-1x filters in a single filter housing with and without separators at 12 mL/min, 22°C.

Methylene blue adsorption breakthrough curves of 400 gsm filters comparing single layer configuration with four layers are presented in Figure 5. Breakthrough curves were obtained under constant flow rate with and without polypropylene separators between layers and on top of the first layer. Flow rate was set such that the flux is matching the flux values from constant pressure experiments for the same samples (12 mL/min).

Adsorption capacity is also much higher with multilayered filters under constant flow mode. The saturation occurred much quicker for both filters without separators. However, adsorption capacity decreased for both type of filters without separators. The capacity decreased by almost 15% for multilayered filter (100 gsm-4x) and 29% for single layer filter (400 gsm-1x) by removing separators. Adsorption capacities are found in Table 1.

**Table 1:** Methylene blue adsorption capacities of single layer and multi-layered filters with and without separators under constant flow rate (12 mL/min).

**Filters**	**Adsorption capacity (mg/g)**
100 gsm-4x-with separators	0.51 ± 0.04
400 gsm-1x-with separators	0.44 ± 0.01
100 gsm-4x	0.43 ± 0.05
400 gsm-1x	0.31 ± 0.05

The offset at the beginning of the curves without separators needs further study to better understand the phenomenon involved.

### 3D X-ray tomography

**Figure 6:**
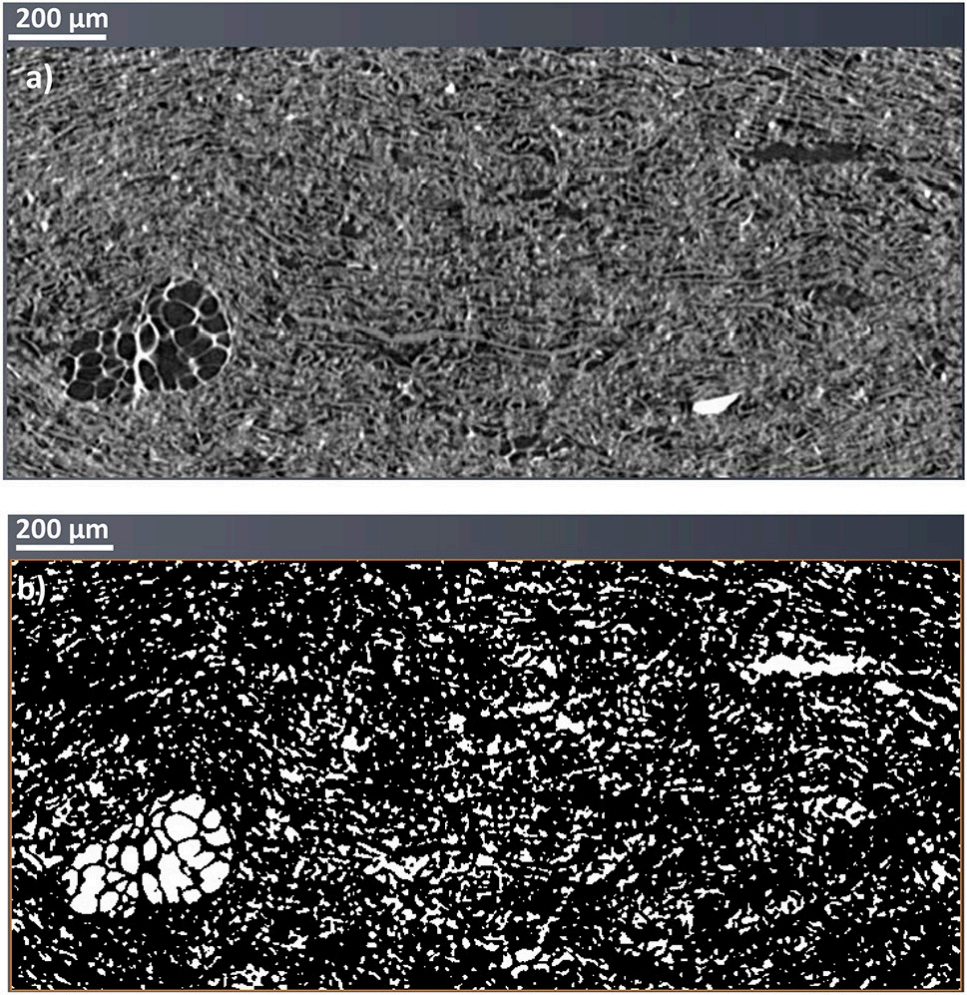
3D X-ray reconstituted images of 400 gsm sample (400 gsm-1x). **(a)** Grayscale and **(b)** Thresholded cross section images.

**Figure 7:**
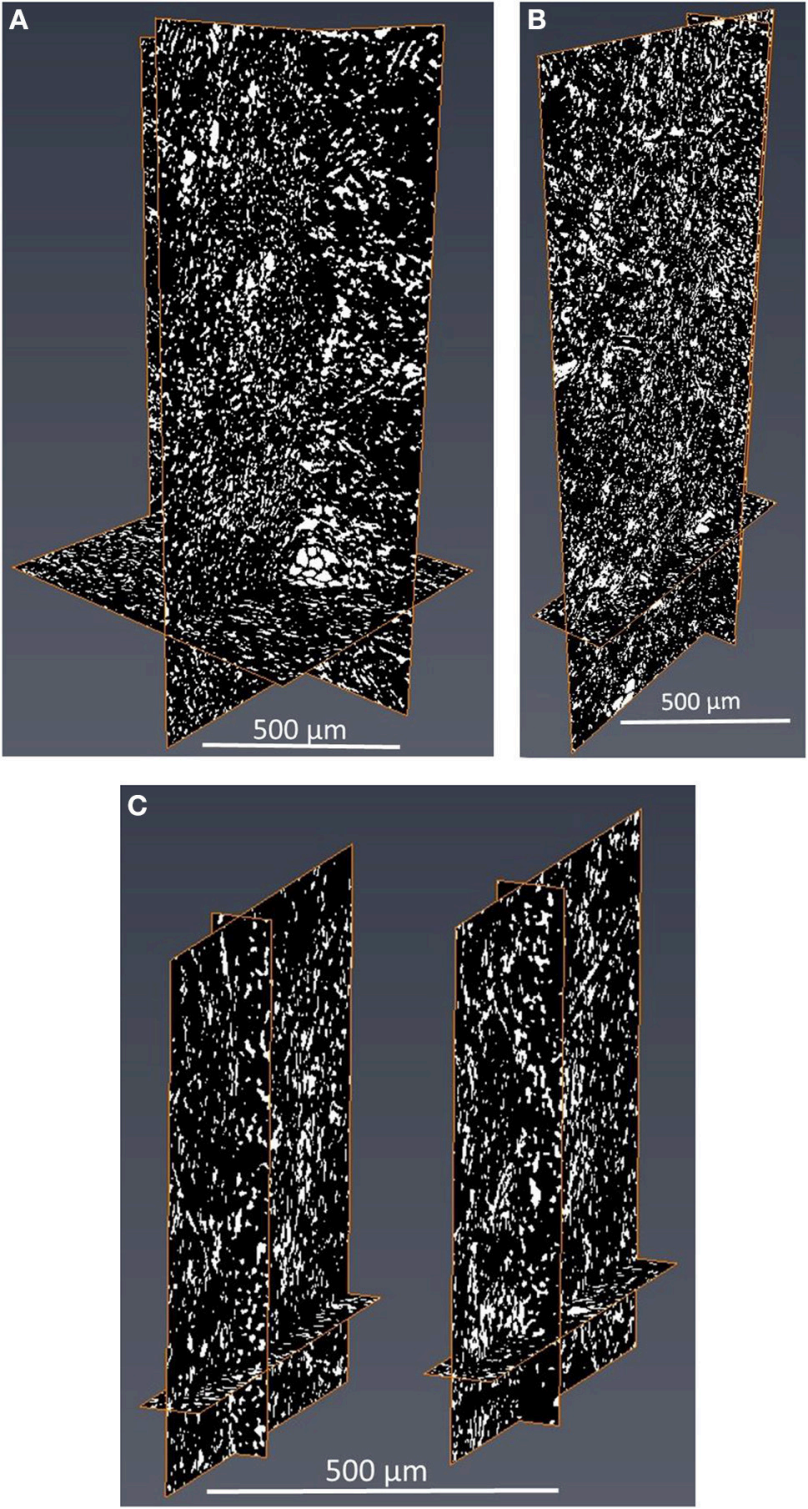
3D X-ray images of **(A)** 400 gsm sample **(B)** 200 gsm sample and **(C)** 100 gsm sample presented with some of layers with intersection of three different planes.

3D X-ray tomography image analysis was conducted on single layer 400 gsm (400 gsm-1x), two layers of 200 gsm (200 gsm-2x) and four layers of 100 gsm (100 gsm-4x). Grayscale and thresholded cross section images of 400 gsm-1x are given in Figure 6. 3D images of all filters are shown with intersection of three different planes (Figure 7). The white sections represent voids in thresholded images. Animations on voids change locations through thickness are provided for all samples in Supplementary information.

The total void fraction of the composites calculated by image analysis is 20% for the 400 and 200 gsm filters and 14% for the 100 gsm filters. However, all void fractions are similar, as expected for filters of identical composition. The main difference lies in the pore structure, which differs with thicknesses, despite having a similar void fraction. The probability of forming bigger voids increases with the composite thickness. There are some zones of low density, creating flow channels in the 400 gsm single layer sample (Figures 6, 7). These channels appear to propagate through the thickness and then to disappear. Channels are best observed in the animations provided in SI. These massive void concentration decreases at lower basis weights, particularly in 100 gsm composites.

**Figure 8:**
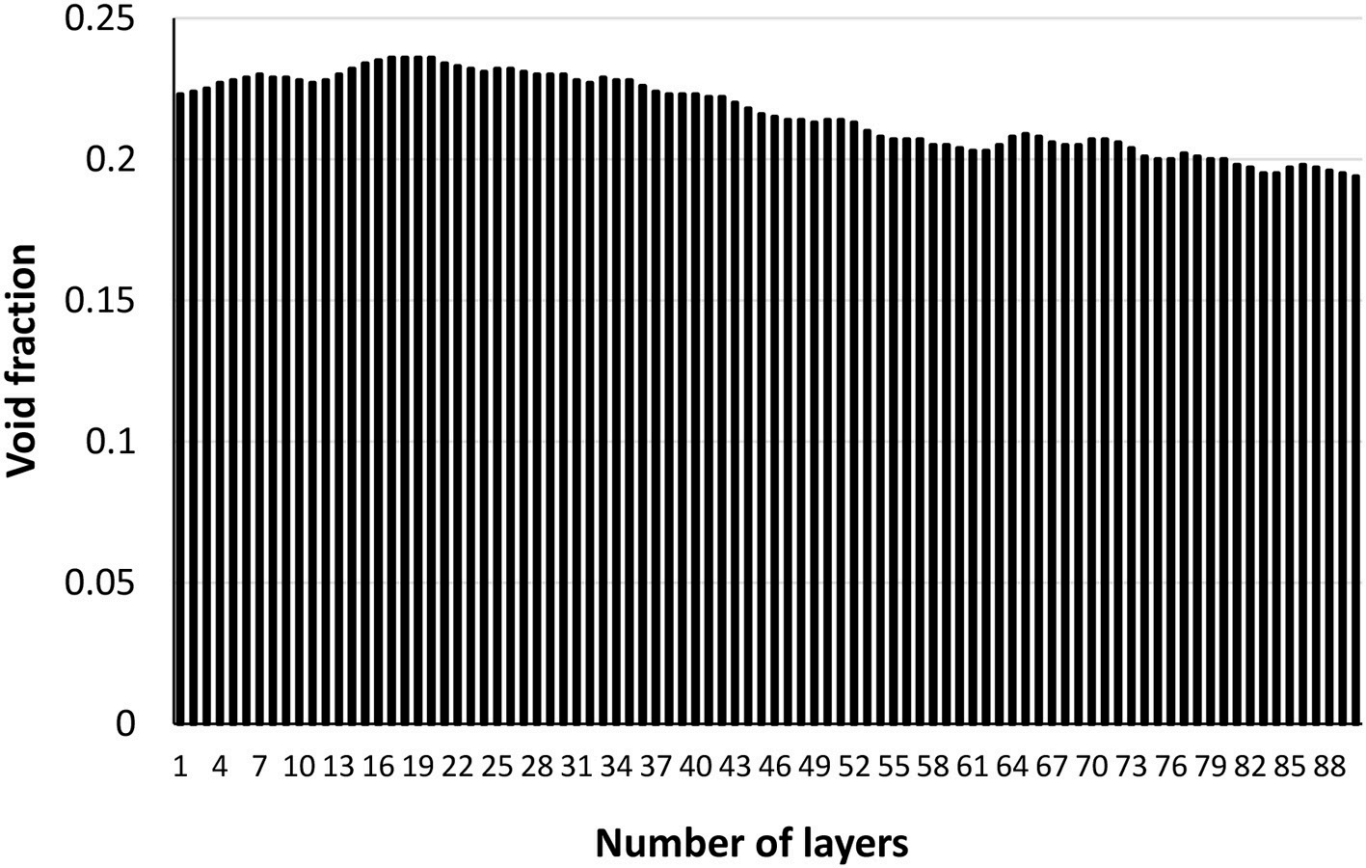
Void fraction distribution through the thickness of an individual layer (200 gsm). The composite mesh side is at the high number (88) and the air side is at the origin.

The void distribution through thickness of an individual layer (200 gsm-2x) is shown in Figure 8. The composite mesh side is at the high number (88) and the air side is at the origin. Void distribution of 200 gsm filter through the thickness is decreasing from top to bottom (Figure 8); this density gradient is inherent to the papermaking technique. The density gradient across filter thickness with the highest composite density (lowest volume fraction) on the mesh side are expected from papermaking. The void distributions for all composites are provided in Supplementary information (Figures S1–S4). However, it was not possible to visualize density gradient in all samples; this suggests that the subtle differences in structure affecting density are below the resolution of the X-ray tomography used. Sample properties based on different gsm were calculated using the properties of fiber and perlite and are shown in Supplementary material in Table S3. The calculated porosities are comparable to the porosities measured by X-ray tomography. However, these calculations suggest that the structure would be slightly denser with the increasing basis weights.

### Filtration

The filtration ability of filters was quantified for the different configurations using polyethylene glycol polymers (PEG) of two molecular weights (600 and 5,000 kDa) and 0.5 μm SiO_2_ colloids. Both PEGs passed through the filters without showing any cut off. Total organic carbon content (TOC) of PEG solutions before and after filtration were identical and given in Supplementary information (Tables S1, S2). These filters are unable to provide microfiltration. The particle rejection of 0.5 μm SiO_2_ particles was then measured. Very high particle rejection was recorded for the 400 and 200 gsm single layer filters. Particle rejection rate however dropped down for 100 gsm two layers (100 gsm-2x) and four layers (100 gsm-4x) filters. This shows that the actual thickness of the layers plays an important role in particle capture, regardless of the number of layers. SiO_2_ particle rejection results are given in Table 2. Besides, 100 gsm-4x filter also shows higher variation in rejection results. This variance potentially shows that multi-layered filters formed by low gsm layers cannot be relied upon for rejecting particles. However, further studies on filtration are needed to fully characterize the mechanism behind the rejection capability of multi-layered filters.

**Table 2:** 0.5 μm SiO_2_ particle rejection of 400 gsm-1x, 200 gsm-1x, 100 gsm-2x, and 100 gsm-4x filters.

**Filters**	**SiO_2_ rejection (%)**
100 gsm-4x	87 ± 11
100 gsm-2x	87 ± 3
200 gsm-1x	98 ± 0.9
400 gsm-1x	99 ± 0.2

## Discussion

### Effect of operation modes: constant pressure or constant flow

Under constant flow, pressure drop builds up across the filter to maintain the flow as fouling occurs over time (Goldrick et al., 2017). However, during constant pressure operations, flux decreases gradually as the filter fouls (Goldrick et al., 2017) and initial high flux in constant pressure mode can result in severe fouling in processes with suspended particles. High flux at the beginning of filtration causes much faster deposition of particles on the membrane surface than are back transported, resulting in faster particle deposition than in constant flow rate mode (Yoon, 2016). This reduces the overall capacity of the filter. Therefore, industrial processes are mainly operated under constant flow mode to maximize the available filter area by avoiding sudden fouling. However, filtering solute molecules can be different with constant pressure as there is no particle involved; our experiments are conducted with solute dye molecules and the main mechanism for separation is by electrostatic adsorption.

During our constant pressure adsorption experiments with multilayer filters, flow rate decreased compared to single layer as a result of the resistance created by the slightly increased thickness of multi-layers. Decreased flow rate results in an increased residence time for liquid, which provides more time for dye molecules to adsorb. In contrast, residence time does not change for multi-layers under constant flow as flow rate is constant. This explains why adsorption capacities at a given basis weight is so dependent upon layer configuration under constant pressure mode.

### Effect of multi-layered filter structure

Heterogeneous composite filters combining fibers and adsorbent particles can develop heterogeneity at small length scales, creating channels. This is accentuated by the agglomeration of adsorbent particles and poor fiber distribution (formation). Liquid flows through such channels with little resistance and saturates the surrounding filter medium of this preferential flow path; this reduces the effectiveness of filters substantially.

**Figure 9:**
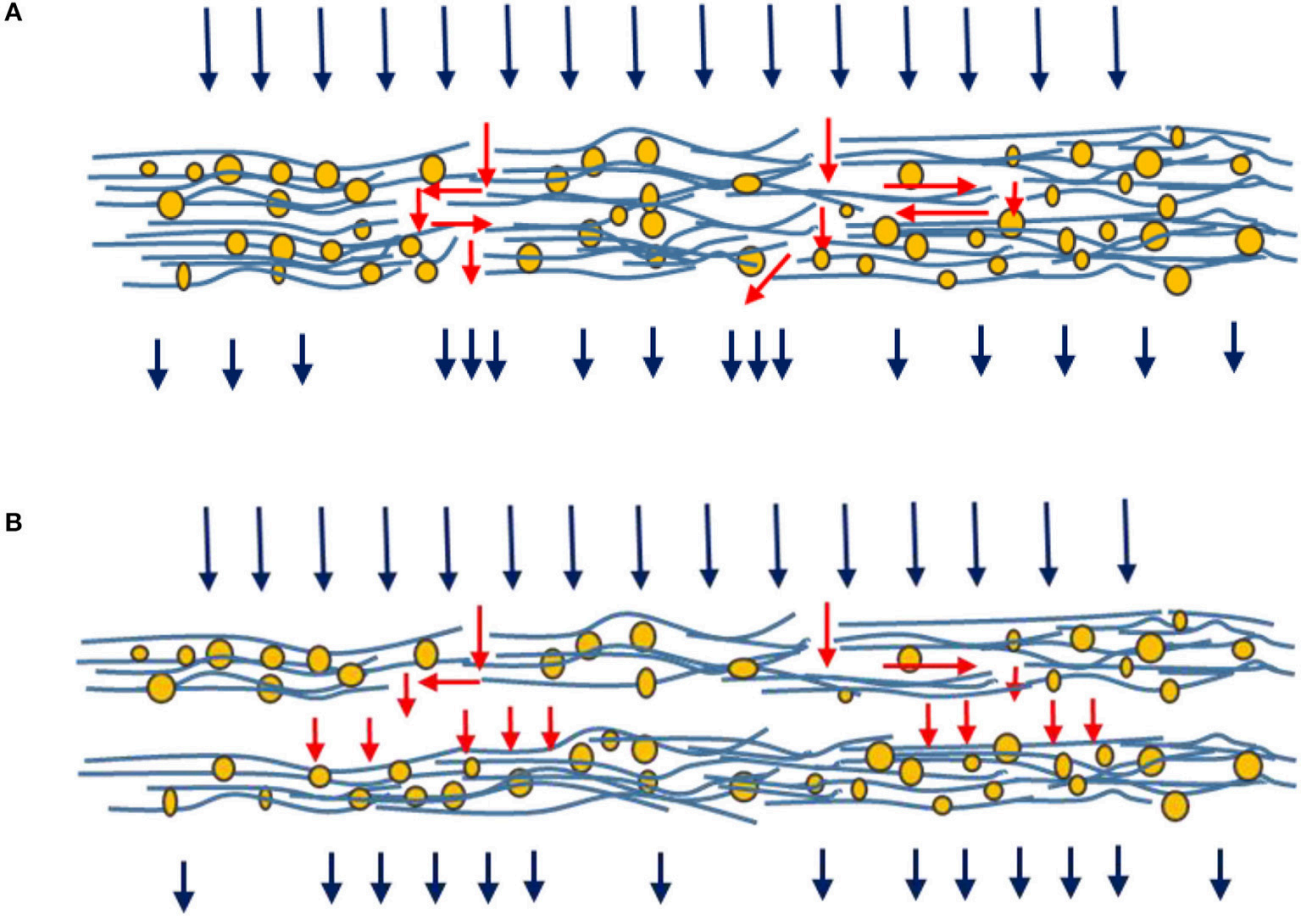
Schematic illustration of flow through a single layer **(A)** and two layers **(B)** filters.

By introducing multi-layers, tortuosity is increased and channeling mostly avoided as multi-layers offsets the channel alignment between consecutive layers. This prevents preferential flow through continuous channels across the entire filter medium. Multi-layers have also increased external surface area maximizing filter-liquid contact (Figure 9).

Figure 9 shows liquid streams following the tortuous path of least resistance in a single layer structure (A). This pattern is distorted between two stacked layers as liquid contacts the filtering medium of the next layer (B). This explains the improved breakthrough curves as a result of operating multi-layered filters for the same basis weight.

Analysis of 3D X-ray tomography images and animations (SI) reveals that a single thick layer of filter (400 gsm-1x) is more likely to contain macro voids than thinner filters; these massive gaps are absent at lower basis weights. Macro voids are defects reported in literature mainly for membranes prepared by phase-inversion methods. The presence of such macro voids results in compaction or collapse of the membranes that reduces flux (Paulsen et al., 1994; Kosma and Beltsios, 2012). In our case, these defects cause preferential liquid flow through these gaps in single layer that results in a poor adsorption capacity. However, the filtering efficiency decreases with multiple layers of thinner filter layers (100 gsm-4x and 100 gsm-2x). Thicker depth filters can better hold particles and prevent any particle escape; filter density gradient across the thickness can be an explanation. Usually, cellulosic fiber composites are formed by wet papermaking laying technique with a density gradient across the thickness. The density increases from top to bottom. The decreasing void fraction through the thickness can be seen in Figure 8. This is due to the preferential distribution of fines away from the mesh and their varying degrees of compaction through the sheet thickness (Rosenthal et al., 1977). Thick single layer filters are expected to have a higher density variance through thickness than smaller basis weights. Also, thick samples are expected to have higher densities than low basis weight samples (the density and porosity calculations based on thickness of the samples are given in the Supplementary section). Another explanation could be the denser structure of thick samples better trapping particles.

### Effect of separators

**Figure 10:**
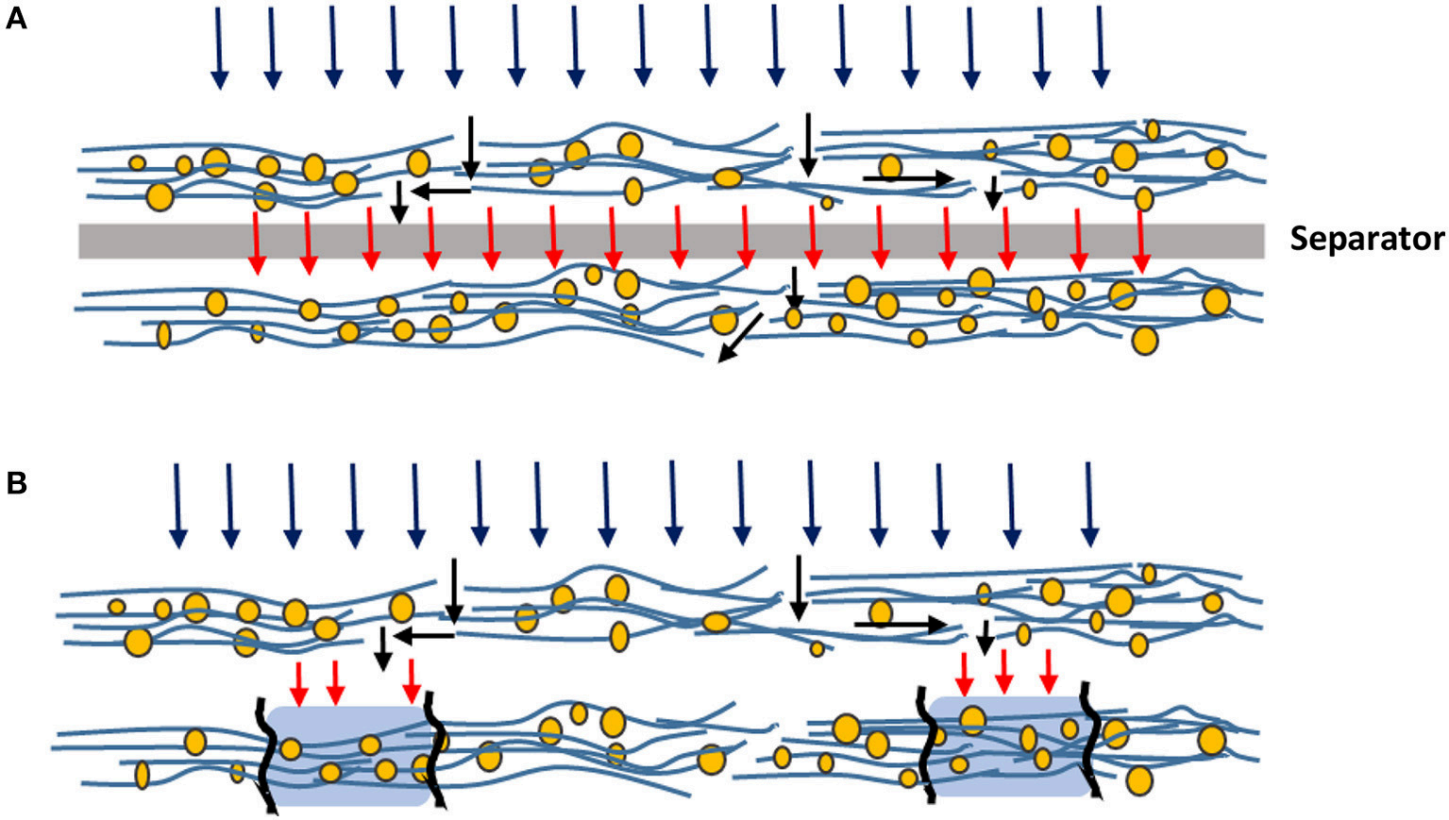
Schematic illustration of flow of liquid through filters with **(A)** and without **(B)** separators.

The improved adsorption capacity with separators under constant flow operation is attributed to a uniformly distributed flow between the layers. Well-distributed flow increases the probability of liquid to follow through the entire filter medium instead of following some preferential pathway and saturating local areas. The operations schematic is illustrated with and without separators in Figure 10.

### Perspective

A thermal process, pasteurization is usually used for food preservation in the food industry. Treatment temperature ranges from less than one minute up to several minutes at temperatures varying from 100 to 150°C, based on products (Lopes et al., 2016). However, this process can alter the organoleptic characteristics and degrade the quality of food, specifically in heat-sensitive foods such as juices and wine (Lopes et al., 2016). Removal of microbial spoilage from liquid foods at low temperatures through filtration is a promising method for food and beverage industry (Papafotopoulou-Patrinou et al., 2016). In this work, we developed food grade filters made from cellulose, a naturally abundant, biocompatible and low-cost material. These filters can remove spoilage as small as 0.5 μm [in the size range of bacteria (Microbiological hazes and deposits^2^)] by up to 98% with 200 gsm two layers and 400 gsm single layer and at least by 87% rejection rate with 100 gsm two and four layers. Filtration can also serve as a preliminary step before any further treatment—including pasteurization (Tomasula et al., 2011; Wray, 2015). The contaminants smaller than the pore size also retained through adsorption combining electrostatic interactions; these interactions are further improved via multi-layer filter configurations. Depth filters can provide a good alternative for “cold” pasteurization process.

## Conclusion

The effect of multi-ply and inter-ply spacing of novel inorganic-cellulosic depth filters was investigated in terms of adsorption capacity and filtration efficiency. Cellulose fiber-perlite composite layers were prepared at three different thicknesses (basis weights) using a papermaking technique. Perlite and fiber content was maintained at 70 and 30 wt.%, respectively. Polyamide-amine-epichlorohydrin (PAE) was used for wet strength, filler retention and charge control. Filters were constructed as single-layer and multi-layers for different total basis weights. Methylene blue (MB) was selected as a cationic solute molecule to characterize adsorption under constant pressure and constant flow rate modes for the different filter configurations. Filtration efficiency was measured with PEG molecules of two molecular weights (600 and 5,000 kDa) and 0.5 μm silicon dioxide (SiO_2_) particles. The filter structure was quantified by 3D X-ray tomography.

MB adsorption capacity increased by multi- layered filter structures at a given basis weight for both operation modes (constant pressure/flowrate). The constant pressure mode is a better option for multi-layer filters as it maximizes adsorption capacity. The adsorption capacity of multi-layered filters (over single layer) increased by preventing flow channeling by off-setting macro voids-channels through the thickness of the filter; these local heterogeneities are often inherent to papermaking. The probability of forming continuous macro voids-channels through the full thickness of paper sharply decreases as the number of layers increases. Distorted channel alignment by multi-layered structure results in an increase in contact surface area that provides a more efficient adsorption. The presence of separators between layers increased adsorption capacity thanks to a well-distributed liquid flow in constant flow mode. Filters had no rejection capability for PEG molecules. The thickest (400 gsm-1x) and half thickness (200 gsm-1x) filter structures filtered 0.5 μm particles at a very high rejection rate (98%). The rejection rate however decreased with multi-layer structures. Multi-layered composites provide better adsorption performances while filtration is best with single thick layers. This work highlights how engineering materials and operation can improve and change filtration and adsorption performance in filters. A novel generation of multi-layer depth inorganic-cellulose filters can provide new avenues for purification of temperature sensitive suspensions such as food and pharmaceutical streams. However, further studies are still required to fully characterize the filtration mechanisms of multi-layered filters.

## Author contributions

AO carried out the experiment and wrote the manuscript with the support from AN, WB and GG. AN and WB supervised the project with the leadership of GG.

### Conflict of interest statement

The authors declare that the research was conducted in the absence of any commercial or financial relationships that could be construed as a potential conflict of interest.

## Footnotes

^1^CFR–Code of Federal Regulations Title 21. Available online at: https://www.accessdata.fda.gov/scripts/cdrh/cfdocs/cfcfr/cfrsearch.cfm?fr=176.170.

^2^Microbiological hazes and deposits. Available online at: https://www.awri.com.au/industry_support/winemaking_resources/fining-stabilities/hazes_and_deposits/microbiological/.)]
