# Supplementary material for: Multi-Layer Filters: Adsorption and Filtration Mechanisms for Improved Separation
Source: Front Chem. 2018 Sep 12;6:417. doi: 10.3389/fchem.2018.00417 (PMC6143674; doi:10.3389/fchem.2018.00417)
Supplement: Supplementary file 1 [file Table_1.DOCX]

***Supplementary Material***

**Multi-Layer Filters: Adsorption and Filtration Mechanisms for Improved Separation**

**Aysu Onur^1^, Aaron Ng^2^, Warren Batchelor^1^, Gil Garnier^1*^**

^1^ Bioresource Processing Research Institute of Australia, Chemical Engineering Department, Monash University, Clayton, Australia

^2^ 3M Australia, Sydney, Australia

*** Correspondence:** Gil Garnier: [gil.garnier@monash.edu](mailto:gil.garnier@monash.edu)

**1. Void Fraction Distribution of Samples through Thickness**

**Supplementary Figure 1.** Void Fraction Distribution of 400gsm-1x

**Supplementary Figure 2.** Void Fraction Distribution of 200gsm-2x (first layer)

**Supplementary Figure 3.** Void Fraction Distribution of 100gsm-4x (second layer)

**Supplementary Figure 4.** Void Fraction Distribution of 100gsm-4x (third layer)

**3. Polyethylene Glycol (PEG) Filtration**

**Table I:** 600 kDa PEG Filtration TOC Results

| **Sample** | **Analysis** | **Result** |
| --- | --- | --- |
| Control | TOC | TOC:51.92 mg/L TC:52.07 mg/L IC:0.1465 mg/L |
| 200gsm-1x-1 | TOC | TOC:48.98 mg/L TC:49.14 mg/L IC:0.1632 mg/L |
| 100gsm-2x-1 | TOC | TOC:51.92 mg/L TC:52.09 mg/L IC:0.1725 mg/L |
| 200gsm-2x-2 | TOC | TOC:51.59 mg/L TC:51.75 mg/L IC:0.1581 mg/L |
| 100gsm-2x-2 | TOC | TOC:50.57 mg/L TC:50.74 mg/L IC:0.1684 mg/L |
| 400gsm-1x-1 | TOC | TOC:49.18 mg/L TC:49.37 mg/L IC:0.1936 mg/L |
| 400gsm-1x-2 | TOC | TOC:52.46 mg/L TC:52.69 mg/L IC:0.2297 mg/L |
| 200gsm-2x-1 | TOC | TOC:53.05 mg/L TC:53.23 mg/L IC:0.1872 mg/L |
| 200gsm-2x-2 | TOC | TOC:49.29 mg/L TC:49.47 mg/L IC:0.1843 mg/L |
| 100gsm-4x-1 | TOC | TOC:52.59 mg/L TC:52.77 mg/L IC:0.1759 mg/L |
| 100gsm-4x-2 | TOC | TOC:50.76 mg/L TC:50.96 mg/L IC:0.1971 mg/L |

**Table II:** 5000 kDa PEG Filtration TOC Results

| **Sample** | **Analysis** | **Result** |
| --- | --- | --- |
| Control | TOC | TOC:53.97 mg/L TC:54.16 mg/L IC:0.1851 mg/L |
| 200gsm-1x-1 | TOC | TOC:51.70 mg/L TC:51.91 mg/L IC:0.2024 mg/L |
| 200gsm-2x-2 | TOC | TOC:49.80 mg/L TC:50.00 mg/L IC:0.2013 mg/L |
| 100gsm-2x-1 | TOC | TOC:52.19 mg/L TC:52.41 mg/L IC:0.2171 mg/L |
| 100gsm-2x-2 | TOC | TOC:50.87 mg/L TC:51.12 mg/L IC:0.2446 mg/L |
| 400gsm-1x-1 | TOC | TOC:51.76 mg/L TC:52.05 mg/L IC:0.2954 mg/L |
| 400gsm-1x-2 | TOC | TOC:51.96 mg/L TC:52.20 mg/L IC:0.2414 mg/L |
| 200gsm-2x-1 | TOC | TOC:52.62 mg/L TC:52.82 mg/L IC:0.1989 mg/L |
| 200gsm-2x-2 | TOC | TOC:53.61 mg/L TC:53.80 mg/L IC:0.1841 mg/L |
| 100gsm-4x-1 | TOC | TOC:52.41 mg/L TC:52.58 mg/L IC:0.1728 mg/L |
| 100gsm-4x-2 | TOC | TOC:52.18 mg/L TC:52.32 mg/L IC:0.1471 mg/L |

**Calibration curves**

**Supplementary Figure 5.** Methylene blue and silicon dioxide calibration curves


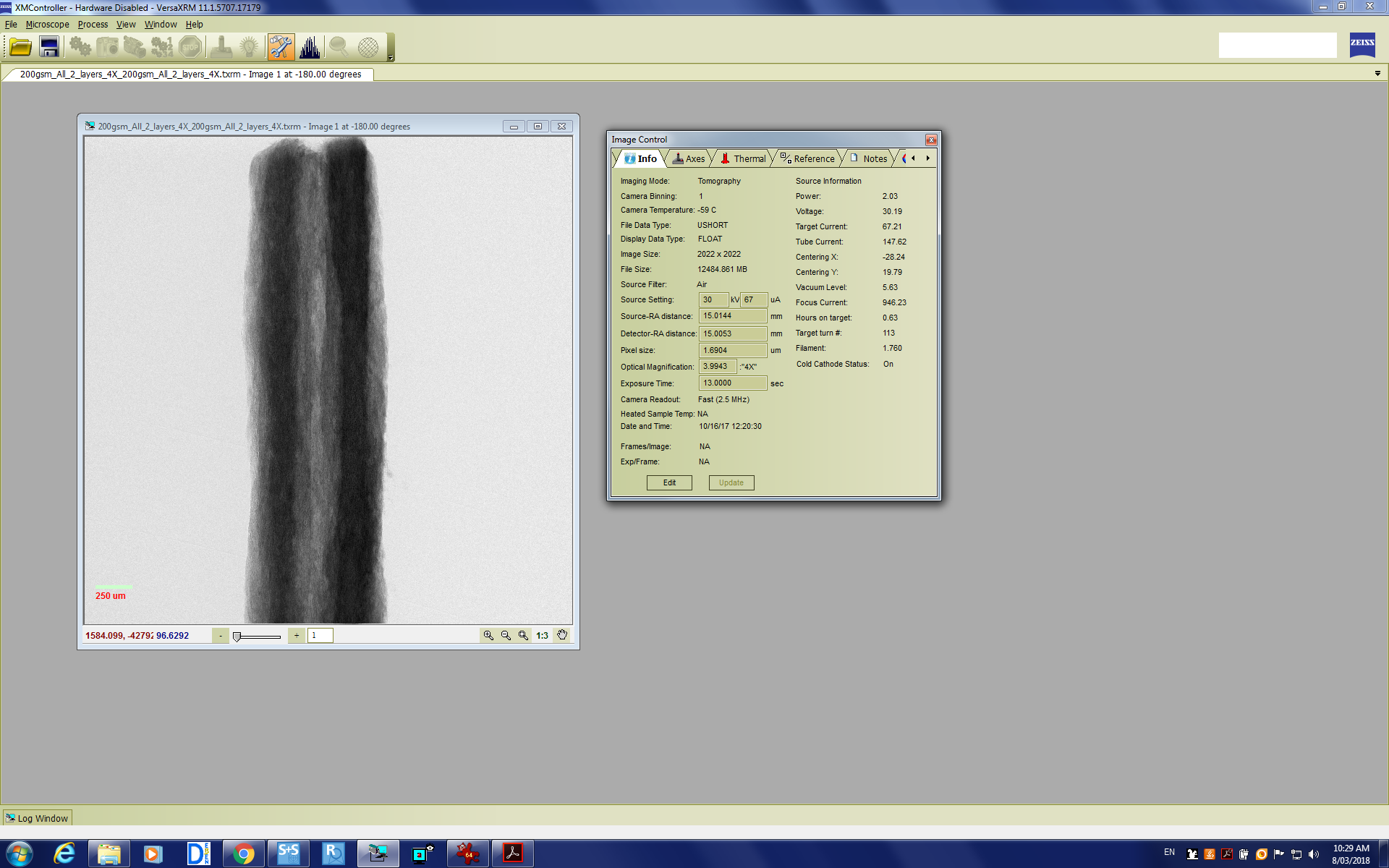


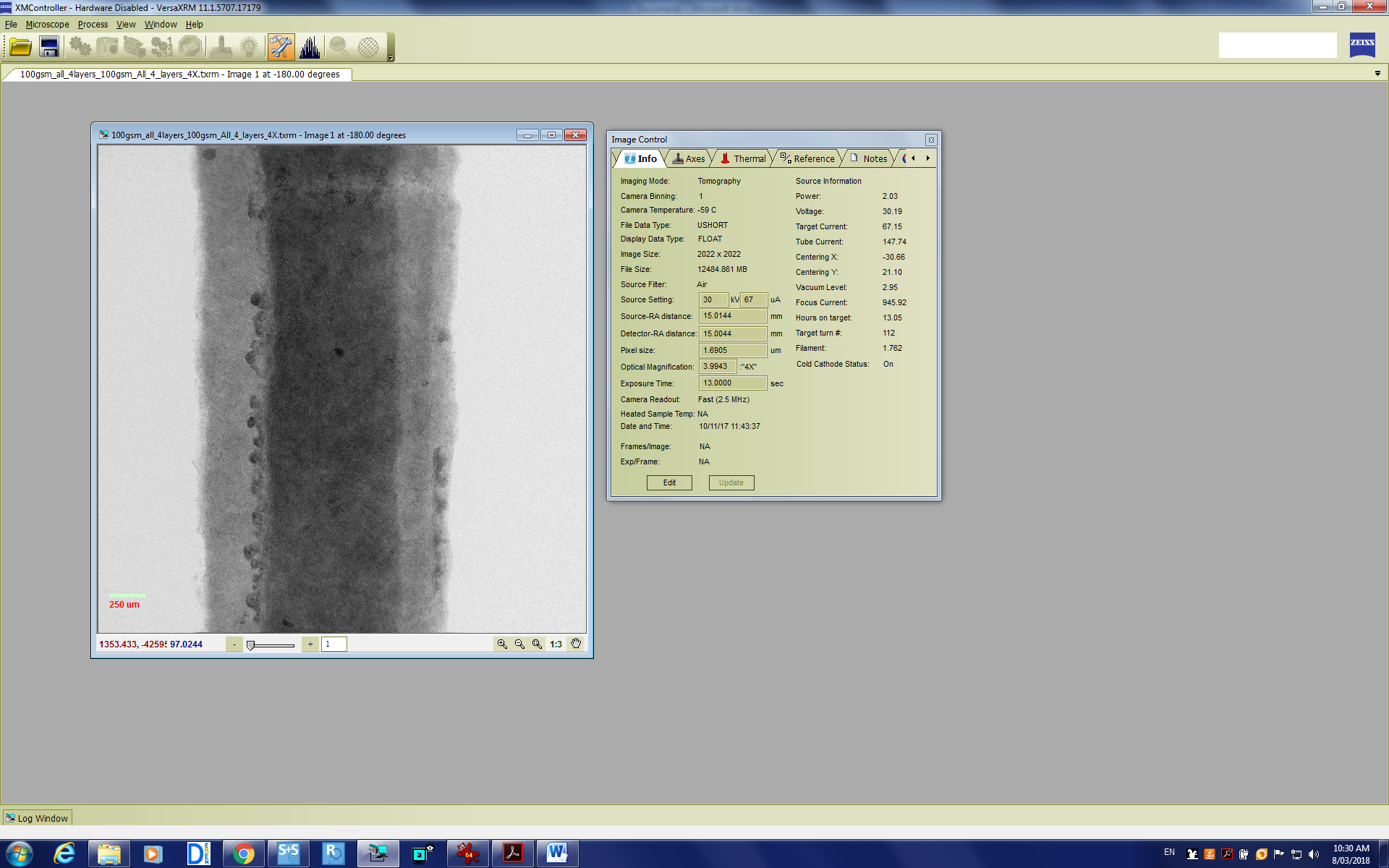


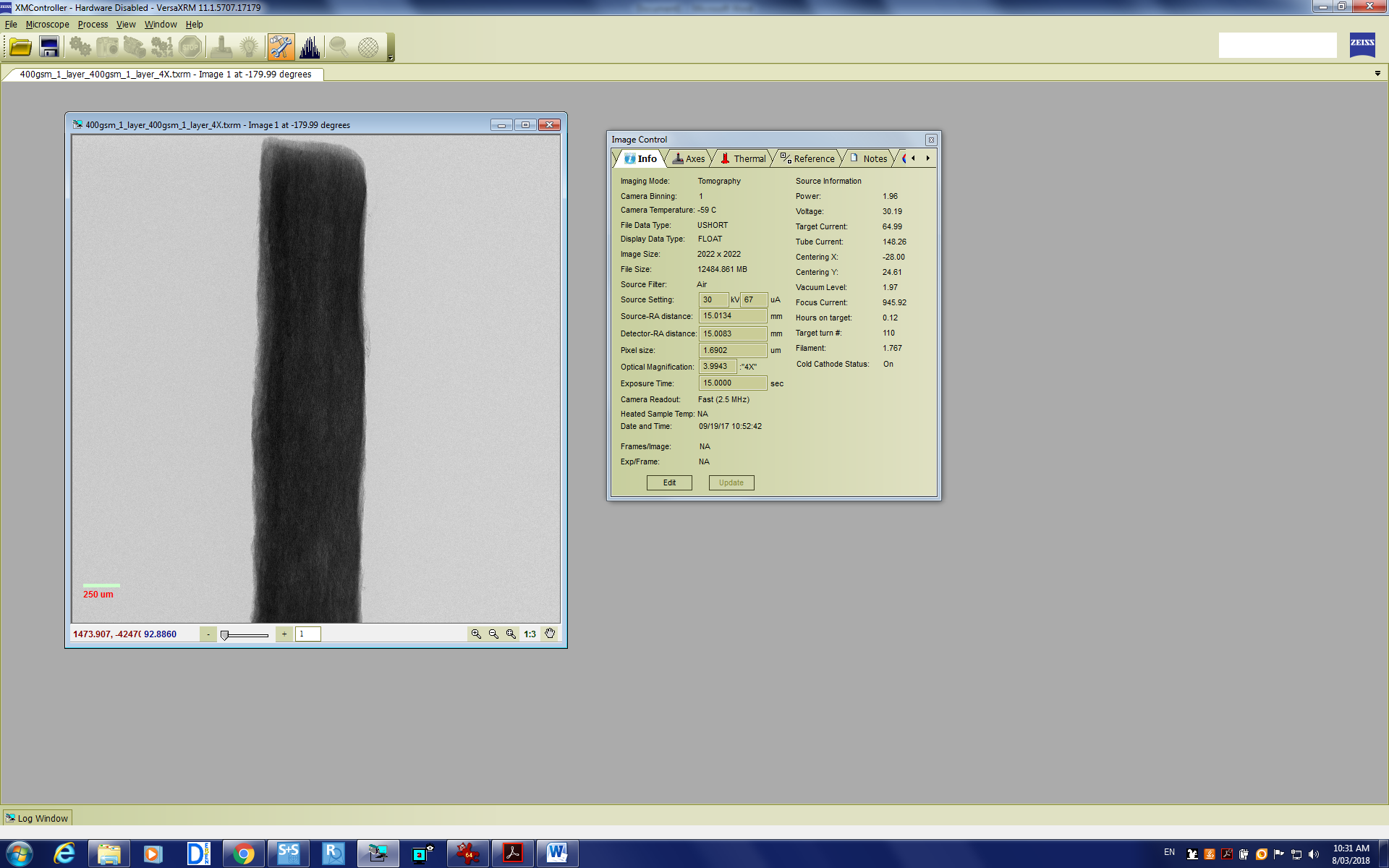


**Supplementary Figure 6.**  3D X-ray scanning and image analysis settings of 200gsm-2x, 100gsm-4x and 400gsm-1x, respectively

**Table III:** Properties of samples based on different gsm

|  | **100 gsm** | **200 gsm** | **400 gsm** |
| --- | --- | --- | --- |
| **Thickness (mm)** | 0.224 | 0.419 | 0.7869 |
| **Weight (g)** | 0.47 | 0.93 | 1.87 |
| **Theoretical density (g/cm^3^)** | 0.555 | 0.555 | 0.555 |
| **Bulk Density (g/cm^3^)** | 0.446 | 0.477 | 0.508 |
| **Porosity (%)** | 0.20 | 0.14 | 0.08 |
| **Flux (LMH)** | 711 | 683 | 574 |
